# The causal effects between low back pain and cerebrospinal fluid metabolites: a two-sample Mendelian randomization study

**DOI:** 10.1186/s41065-025-00374-y

**Published:** 2025-02-07

**Authors:** Run Peng, Xiaoxin Wang, Wei Wang, Zeqin Li, Yuze Sun, Mingliang Yang

**Affiliations:** 1 School of Rehabilitation Medicine, Capital Medical University, Beijing, China; 2 Department of Spinal and Neural Functional Reconstruction, China Rehabilitation, Research Center, Beijing, China; 3 Center of Neural Injury and Repair, Beijing Institute for Brain Disorders, Beijing, China; 4 Beijing Key Laboratory of Neural Injury and Rehabilitation, Beijing, China

**Keywords:** Cerebrospinal fluid metabolites, Biomarkers, Low back pain, Mendelian randomization, Causal relationship

## Abstract

**Background:**

Observational studies have shown an association between cerebrospinal fluid (CSF) metabolites and low back pain (LBP), but the causal relationship between these factors remains unclear.

**Methods:**

We performed a two-sample Mendelian randomization (MR) analysis to examine whether there is a causal relationship between CSF metabolites and LBP. We applied several MR methods, including inverse variance weighting, weighted median, MR-Egger, Wald ratio, and MR-PRESSO, to test the causal relationship and conducted a sensitivity analysis to assess the robustness of the results.

**Results:**

We identified a total of 12 CSF metabolites significantly associated with LBP, of which Bilirubin, 5,6-dihydrothymine, Erythronate, Mannitol/sorbitol, and Butyrate have a potential inhibitory causal effect on LBP risk (*p* < 0.05). Meanwhile, 2-hydroxyadipate, Gamma-glutamyl-alpha-lysine, Indoleacetate, N-acetylputrescine, Palmitoyl dihydrosphingomyelin, S-methylcysteine, and 2,3-dihydroxy-5-methylthio-4-pentenoate play a causal role in increasing the risk of LBP (*p* < 0.05). No significant estimates of heterogeneity or pleiotropy were detected.

**Conclusion:**

Our study emphasizes the causal relationship between CSF metabolites and LBP risk, providing reference for clinical treatment and prognosis of LBP.

**Supplementary Information:**

The online version contains supplementary material available at 10.1186/s41065-025-00374-y.

## Introduction

Low back pain (LBP) is the leading cause of disability globally and one of the most common musculoskeletal disorders, affecting people of all ages across countries with different income levels [1, 2]. According to its etiology, low back pain can be divided into specific and non-specific categories, with non-specific low back pain accounting for about 80–90% of all cases [3]. According to the Global Burden of Disease (GBD) study, as the population ages, the prevalence of LBP and the years of life lost to disability due to LBP rise with age, reaching their peak at around 85 years old [4]. The average prevalence of low back pain in the general adult population is approximately 12%, with higher rates in individuals aged 40 and older, as well as in women; the lifetime prevalence is about 40% [5]. Additionally, LBP leads to 69 million years lived with disability (YLDs), accounting for 8.1% of all YLDs, making it the leading cause of YLDs and the primary cause of global disability. It is projected that the number of cases will reach 843 million by 2050 [4]. Moreover, studies from countries such as the UK, Australia, and the US have shown that the societal costs of LBP, particularly productivity loss, lead to a substantial economic burden [6, 7]. As a result, LBP not only severely impacts patients’ quality of life, consumes substantial healthcare resources, but also leads to increased healthcare costs and productivity losses, creating a heavy burden on individuals, families, and society [8].

Metabolites, acting as functional intermediates, can reveal the relationship between genetic variations and metabolites, thereby assisting in understanding the biological mechanisms of human diseases.Alterations in metabolites could represent a risk factor for disease.The pathophysiological mechanisms of LBP remain unclear, especially for non-specific low back pain.In recent years, biomarkers, which can objectively measure and evaluate general biological processes, disease progression, or drug responses related to medical interventions, have gained significant attention.Despite prior research summarizing some biomarkers related to LBP, several plasma inflammatory markers are considered key substances that contribute to the development of LBP [9, 10]. Zwart et al. [11] used proton nuclear magnetic resonance spectroscopy to analyze various metabolites in human cerebrospinal fluid, finding that LBP or sciatica patients exhibited higher metabolic activity levels compared to pain-free control subjects, with significantly lower levels of several key metabolites, especially in patients with disc herniation or spinal cord imaging abnormalities. A study found that 13 blood metabolites were causally related to the risk of LBP caused by intervertebral disc degeneration (IVDD), with 11 showing a negative correlation and 2 showing a positive correlation [12]. This suggests that plasma metabolites or cerebrospinal fluid metabolites may be associated with the occurrence of LBP.However, the current evidence is not sufficient to directly associate these metabolic changes with pain severity or the activity status of LBP over time.Moreover, these studies are mostly observational, which inherently limits their conclusions. On the one hand, the sample sizes are often small; on the other hand, biomarkers related to LBP tend to have low sensitivity and specificity, and are easily affected by disease covariates like age, BMI, and depression.Crucially, observational studies cannot address the reverse causality effect.For example, do different types of LBP lead to changes in plasma metabolites, or are changes in plasma metabolite concentrations a cause of LBP? It remains unclear whether LBP causes changes in plasma metabolite concentrations, or if the change in plasma metabolite concentrations triggers** LBP**.

Therefore, using Mendelian randomization(MR) to determine the relationship between LBP and exposure factors has significant advantages. Previous studies have found that 13 blood lipid metabolites and immune cells in blood have a causal relationship with the risk of LBP caused by intervertebral disc degeneration (IVDD) [12, 13]. Furthermore, Modic changes (MC) in the lumbar spine are strongly correlated with the development and severity of LBP, and causal relationships suggest a significant connection between serum lipid metabolites and MC [14]. However, most studies aimed at identifying biomarkers for LBP have concentrated on the causal relationship between plasma metabolites and LBP [9, 12, 15, 16]. The causal link between cerebrospinal fluid (CSF) metabolites and LBP remains uncertain, and there are currently limited studies on this relationship.

MR is a causal inference method based on genetic variation that can assess the causal relationship between risk factors and disease occurrence. Since genetic variation is randomly distributed during meiosis [17], it mimics a clinical randomized controlled trial without requiring the vast human and material resources typically needed for such experiments [18]. In addition, it employs multiple sensitivity analyses to exclude the impact of confounding factors on causality [19], effectively addressing reverse causality [20], which is one of its advantages over observational studies [21].

Therefore, we used MR to analyze the GWAS data of LBP and 338 cerebrospinal fluid metabolites to assess the causal effect of cerebrospinal fluid metabolites on LBP risk. We aim to integrate metabolomics and genomics by identifying the metabolic pathways mediating LBP, with the goal of providing new insights into early diagnosis and treatment strategies for LBP.

## Methodology

### Study design 

**Fig. 1:**
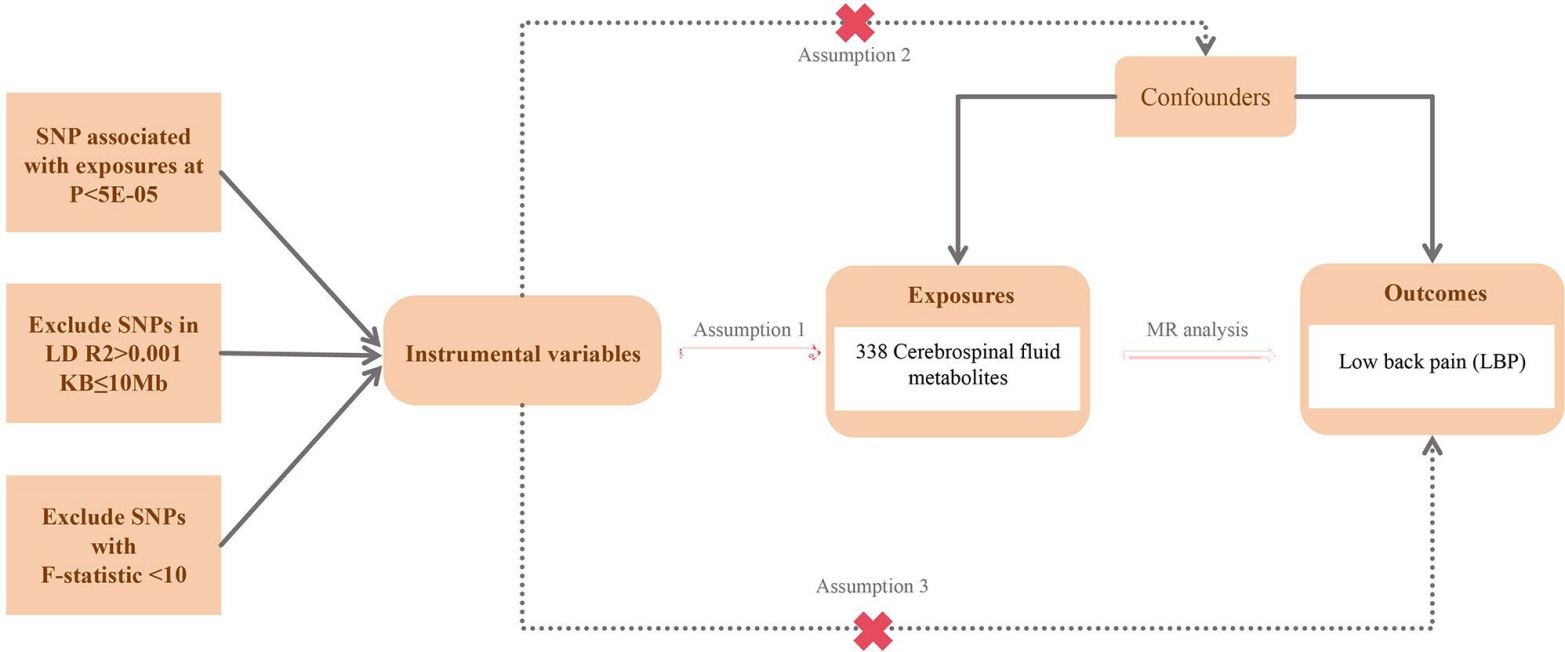
The flowchart of the study

The detailed analysis process of this study is illustrated in Fig. 1. MR should be conducted under three key assumptions: (1) genetic variations are closely associated with the exposure; (2) genetic variations are independent of any known or unknown confounding factors; (3) genetic variations affect the outcome solely through exposure, not via any other direct causal path. This implies that we must control for the pleiotropy of genetic variations and issues such as linkage disequilibrium. The design of this study uses 338 cerebrospinal fluid metabolites as exposures and low back pain (LBP) as the outcome to evaluate the effect of changes in cerebrospinal fluid metabolites on LBP.

### GWAS summary data on metabolites

The metabolite GWAS summary data were sourced from the NHGRI-EBI GWAS Catalog. The GWAS sample includes 291 adults from Europe, and around 7.05 million SNPs from this population were analyzed for association. After quality control, 338 metabolites were included in the GWAS analysis. Detailed data information can be found in Supplementary Table 1.

### GWAS summary data for LBP

The GWAS data for LBP were obtained from the FinnGen biobank (DF10 - December 18, 2023), and all data can be accessed at https://www.finngen.fi/en.The FinnGen study is a large-scale genomic initiative that analyzed over 500,000 Finnish biosamples and their associated genetic variations. Summary statistics can be freely downloaded from the website. All of these data are de-identified, available for free download, and can be used without limitations.

### Selection of instrumental variables

Initially, SNPs closely associated with the exposure were selected based on their genome-wide significance in the GWAS (*P* < 1 × 10 − 5). The clumping distance was > 10,000 kb, and the linkage disequilibrium level (r2 < 0.001) was applied. To assess whether the retained SNPs might be subject to weak instrument bias, the F-statistic was used and calculated. SNPs with an F-statistic less than 10 were considered weak instruments and excluded [22]. The F statistic > 10 will enhance the robustness of the results and reduce the risk of weak instrument bias. After excluding weak instrumental variables from the exposure and outcome datasets, the remaining SNPs were used for MR analysis.

### MR analysis

We used the most commonly used MR method—the inverse variance weighted (IVW) method—to assess the causal relationship between CSF metabolites and HS. This method can precisely estimate causal effects when all SNPs are valid instruments. We supplemented our validation with MR-Egger regression, weighted median, weighted mode, and simple mode to enhance accuracy and stability. MR-Egger regression considers the intercept term and allows MR analysis in the presence of horizontal pleiotropy (where a single genetic variant affects multiple traits).

The weighted median method takes the median of the weighted instrument variable estimates. It is robust to invalid instruments, providing consistent estimates even if up to 50% of the instruments are invalid. The simple mode method estimates the causal effect based on the distribution pattern of the individual SNP estimate. It assumes that the most common effect estimate is the true causal effect, which is particularly useful when there is heterogeneity among SNPs. This method improves the accuracy of causal effect estimates by weighting more precise SNPs. To ensure consistency and validity of the analysis results, we only included data with consistent OR directions from the five MR methods.

### Reliability assessment

We employed MR-Egger regression to examine whether pleiotropy exists in the IV and whether it affects the results. If the MR-Egger intercept is near 0 or *p* > 0.05, it indicates no pleiotropic effect in the IV [23]. For the IVW method, Cochran’s Q test was used to assess heterogeneity among the IVs [24], A p-value > 0.05 suggests the absence of heterogeneity. *The MR-PRESSO global test was applied to assess whether horizontal pleiotropy is present in the results. To eliminate random errors in IV selection*,* we conducted leave-one-out analysis and single SNP analysis to identify whether specific SNPs affected our results.*

All statistical analyses were conducted in R software (version 4.3.3, R Foundation for Statistical Computing), using R packages including TwoSampleMR (version 0.6.4), ieugwasr (version 1.0.0), gwasglue (version 0.0.0.9), reshape2 (version 1.4.4), circlize (version 0.4.16), ComplexHeatmap (version 2.18.0), grid (version 4.3.3), readr (version 2.1.5), forestploter (version 1.1.2), plyr (version 1.8.9), dplyr (version 1.1.4), MRInstruments (version 0.3.2), data.table (version 1.15.4), pacman (version 0.5.1), BiocManager (version 1.30.23), and gwasvcf (version 0.1.2), etc.

## Results

### MR analysis of CSF metabolites in relation to LBP

The IVW method identified a total of 15 CSF metabolites significantly associated with LBP (*P* < 0.05). These metabolites include 3 from the amino acid metabolism pathway, 1 from the carbohydrate metabolism pathway, 2 from the short-chain organic acid metabolism pathway, 2 from the fatty acid metabolism pathway, 1 from the nucleic acid metabolism pathway, 1 from the heme metabolism pathway, 2 from other specific metabolic pathways (related to gut microbiota metabolism, galactose metabolism products, and metabolites possibly linked to sulfur amino acid metabolism), and 3 unknown metabolites.

**Fig. 2:**
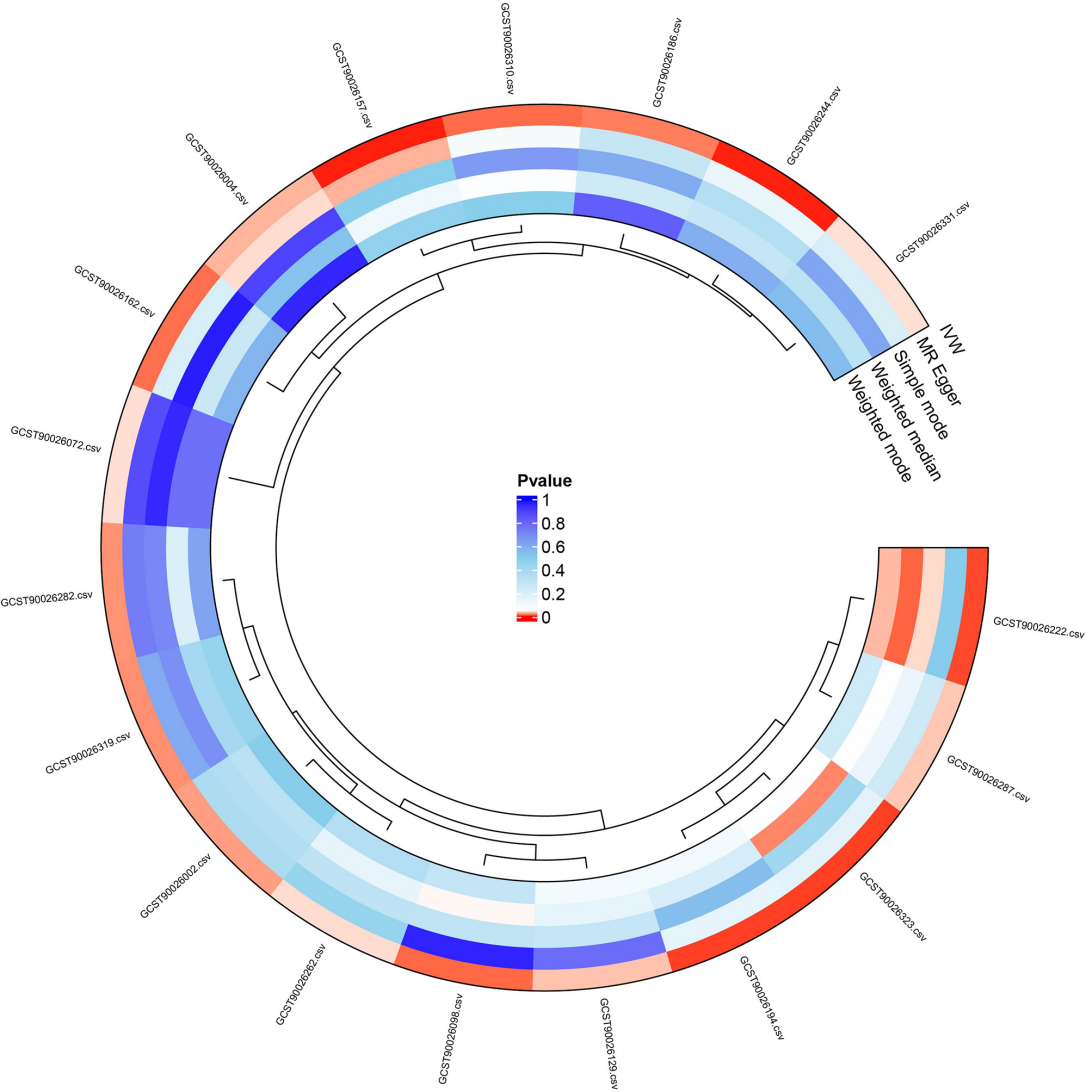
Circular visualization of the causal relationship between CSF metabolites and LBP risk

**Fig. 3:**
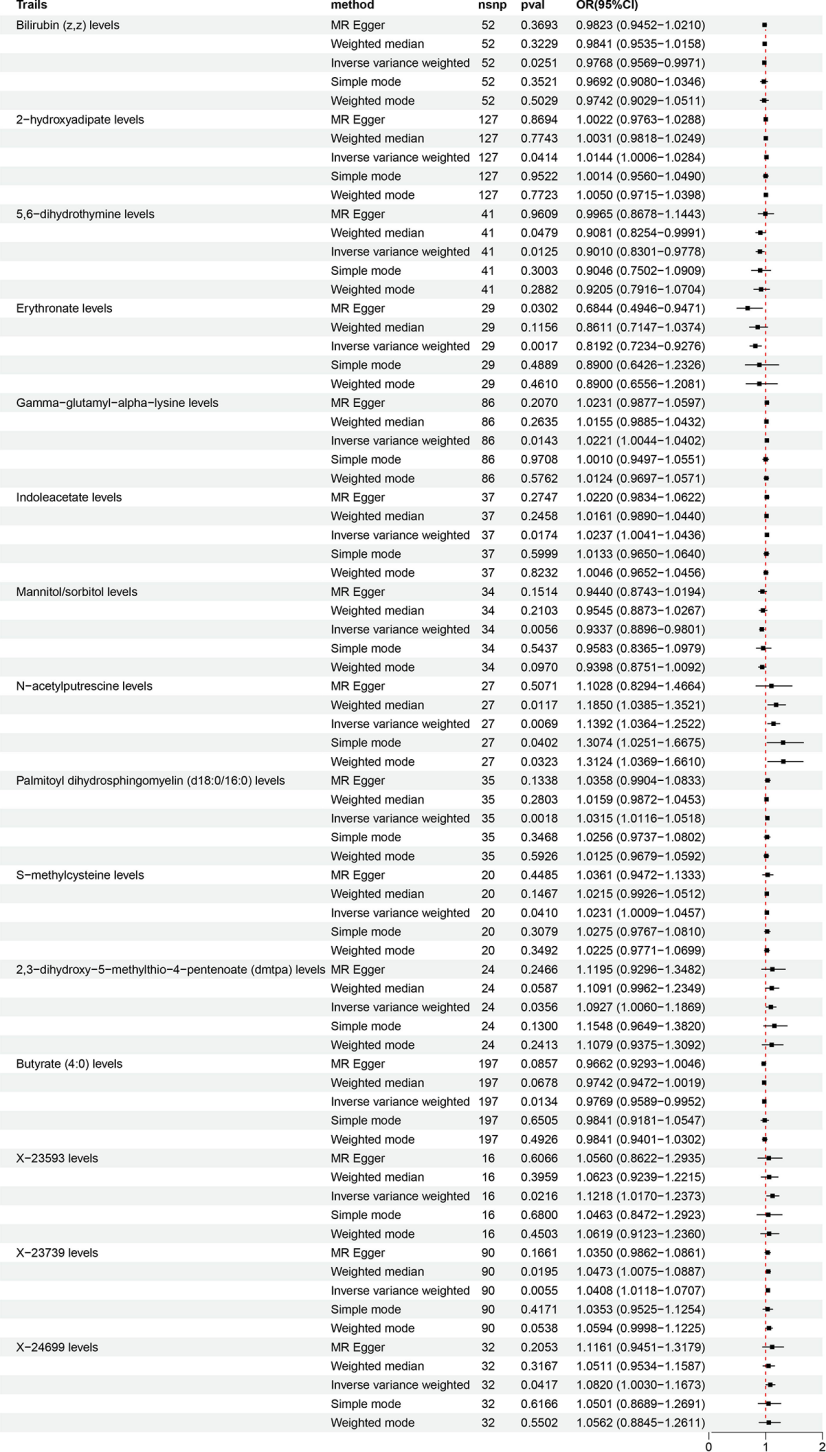
Forest plot of the causal effect of CSF metabolites on LBP risk

This study uses MR analysis to explore the causal relationship between CSF metabolites and LBP. The IVW analysis revealed statistically significant correlations between specific CSF metabolites and LBP, as shown in Fig. 2. Data with consistent OR values from all five MR methods were included, with the forest plot results presented in Fig. 3. MR analysis showed that Bilirubin (z, z) levels (OR = 0.9768, 95% CI: 0.9569 − 0.9971, *P* = 0.0251), 5,6-dihydrothymine levels (OR = 0.9010, 95% CI: 0.8301 − 0.9778, *P* = 0.0125), Erythronate levels (OR = 0.8192, 95% CI: 0.7234 − 0.9276, *P* = 0.0017), Mannitol/sorbitol levels (OR = 0.9337, 95% CI: 0.8896 − 0.9801, *P* = 0.0056), and Butyrate (4:0) levels (OR = 0.9769, 95% CI: 0.9589 − 0.9952, *P* = 0.0134) were inversely associated with the risk of LBP. The levels of 2 − hydroxyadipate (OR = 1.0144, 95% CI: 1.0006 − 1.0284, *P* = 0.0414), Gamma − glutamyl − alpha − lysine (OR = 1.0221, 95% CI: 1.0044 − 1.0402, *P* = 0.0143), Indoleacetate (OR = 1.0237, 95% CI: 1.0041 − 1.0436, *P* = 0.0174), N − acetylputrescine (OR = 1.1392, 95% CI: 1.0364 − 1.2522, *P* = 0.0069), Palmitoyl dihydrosphingomyelin (d18:0/16:0) (OR = 1.0315, 95% CI: 1.0116 − 1.0518, *P* = 0.0018), S − methylcysteine (OR = 1.0231, 95% CI: 1.0009 − 1.0457, *P* = 0.0410), 2,3 − dihydroxy − 5−methylthio − 4−pentenoate (dmtpa) (OR = 1.0927, 95% CI: 1.0060 − 1.1869, *P* = 0.0356), and the other three unknown metabolites all showed a significant positive correlation with the risk of LBP.

### Reliability assessment results

In this study, Cochran’s Q test suggested the presence of heterogeneity in some outcomes. Although PRESSO tests indicated the possibility of horizontal pleiotropy in some outcomes, MR-Egger intercept test showed no evidence of horizontal pleiotropy affecting the relationship between CSF metabolites and LBP (*p* > 0.05) (Table 1). Additionally, leave-one-out sensitivity analysis indicated no single IV had an abnormal impact on the overall results (see supplementary Fig. 1; supplementary Fig. 2), proving the stability of the results.

**Table 1:** The result of heterogeneity test and horizontal pleiotropic test

Exposure	Outcomes	Heterogeneity test				MR-PRESSO	Horizontal pleiotropic test		
		Method	Q	Q_df	Q_pval	P for global test	egger_ intercept	SE	p-value
Bilirubin (z, z) levels	LBP	MR Egger	47.3046	50	0.5822	0.566	-0.0015	0.0045	0.7360
		IVW	47.4196	51	0.6167				
2 − hydroxyadipate levels		MR Egger	149.4223	125	0.0674	0.077	0.0042	0.0040	0.2905
		IVW	150.7694	126	0.0656				
5,6 − dihydrothymine levels		MR Egger	64.0293	39	0.0070	0.003	-0.0090	0.0051	0.0881
		IVW	69.0528	40	0.0030				
Erythronate levels		MR Egger	23.6540	27	0.6494	0.648	0.0070	0.0060	0.2508
		IVW	25.0312	28	0.6261				
Gamma − glutamyl − alpha − lysine levels		MR Egger	90.4649	84	0.2955	0.332	-0.0003	0.0042	0.9524
		IVW	90.4688	85	0.3222				
Indoleacetate levels		MR Egger	23.8684	35	0.9227	0.945	0.0005	0.0055	0.9255
		IVW	23.8772	36	0.9394				
Mannitol/sorbitol levels		MR Egger	36.1861	32	0.2794	0.353	-0.0017	0.0046	0.7176
		IVW	36.3366	33	0.3159				
N − acetylputrescine levels		MR Egger	28.2102	25	0.2983	0.383	0.0017	0.0073	0.8142
		IVW	28.2738	26	0.3451				
Palmitoyl dihydrosphingomyelin (d18:0/16:0) levels		MR Egger	31.8721	33	0.5231	0.572	-0.0012	0.0057	0.8412
		IVW	31.9129	34	0.5703				
S − methylcysteine levels		MR Egger	23.2764	18	0.1802	0.231	-0.0031	0.0109	0.7785
		IVW	23.3819	19	0.2209				
2,3 − dihydroxy − 5−methylthio − 4−pentenoate (dmtpa) levels		MR Egger	26.8024	22	0.2188	0.302	-0.0017	0.0059	0.7769
		IVW	26.9027	23	0.2601				
Butyrate (4:0) levels		MR Egger	261.4166	195	0.0010	0.001	0.0028	0.0045	0.5319
		IVW	261.9422	196	0.0011				
X − 23,593 levels		MR Egger	17.0410	14	0.2540	0.331	0.0046	0.0069	0.5135
		IVW	17.5880	15	0.2849				
X − 23,739 levels		MR Egger	113.6934	88	0.0341	0.03	0.0011	0.0038	0.7764
		IVW	113.7982	89	0.0393				
X − 24,699 levels		MR Egger	43.1524	30	0.0568	0.072	-0.0029	0.0069	0.6832
		IVW	43.3966	31	0.0687				

Q: Cochran’s Q test; Q_df: Degrees of freedom for Cochran’s Q test; IVW: Inverse variance weighted

## Discussion

Over the past few decades, due to the rapid advancements in proteomics and metabolomics, there has been a broad and deep understanding of the pathogenesis and treatment of LBP. However, most studies are animal or case-control studies, which can demonstrate the association between metabolites and cerebrospinal fluid (CSF), but cannot establish causality. Several studies have identified differences in CSF composition between LBP patients and control subjects [25–28], and anatomically, CSF from the spinal canal is located near the intervertebral disc, supporting the validity of this method. Using this approach, Lim et al. reported an elevation of neuroinflammatory markers in the CSF of chronic LBP patients [29]. Compared to healthy controls, protein levels in the CSF of individuals with chronic pain and/or disc degeneration have changed, suggesting that the differences related to disc degeneration and pain are reflected in the CSF. Therefore, identifying factors that may contribute to or alleviate pain could potentially offer new avenues for the treatment of LBP. This study explores the potential causal relationship between cerebrospinal fluid (CSF) metabolites and LBP risk using two-sample Mendelian randomization (MR) analysis, utilizing publicly available summary statistics from the Fengen database. We believe this is the first MR study to systematically evaluate the causal role of human blood metabolites in the pathogenesis of LBP.

Existing observational studies predominantly focus on the relationship between serum inflammatory factors or serum metabolite levels and the risk of LBP [9, 16, 30, 31]. In comparison with serum, CSF has multiple advantages in metabolomics research, particularly in neurological and central nervous system (CNS) diseases. CSF is more directly related to the CNS, less complex, minimally influenced by peripheral factors, and more likely to contain disease-specific biomarkers for CNS disorders [32]. Preclinical studies have confirmed that IL-8 levels are increased in the CSF of chronic LBP patients with intervertebral disc degeneration, and that Reparixin can suppress lumbar pain behavior and disc inflammation in mice33. This indicates that inflammatory factors in CSF could be potential risk biomarkers for chronic LBP and may guide clinical research [33].代Metabolomics research has shown alterations in the metabolic profiles of LBP patients, with the most frequently reported metabolites being amino acids, lipids [34], polyamines, choline, and nucleotides [35, 36]. Kameda et al. [37]. discovered changes in brain metabolites in the anterior cingulate cortex of chronic LBP patients and a correlation between these metabolites and psychological states. Earlier studies indicated no correlation between monoamine metabolites in the CSF of LBP patients and pain. Among the factors studied, height had the greatest influence on the variation in 5-hydroxyindoleacetic acid concentrations, while levels of 3-methoxy-4-hydroxyphenylethanediol increased with age [38], which contrasts with the results of our study. Through a comprehensive analysis of CSF metabolites, this study has further identified key metabolites and metabolic pathways closely associated with the pathogenesis and clinical phenotype of LBP, offering a new perspective for understanding the biochemical foundation of LBP.

This study encompasses a broad range of genetic variables and systematically analyzes the relationship between CSF metabolites and genetic factors associated with LBP in the Finnish database. However, the study also has some limitations. First, the sample size used is relatively small, and further enrichment of metabolite data is required. Secondly, the GWAS database primarily consists of data from European populations, making it unclear whether the findings can be generalized to other ethnic groups. Lastly, due to the limited scope of metabolite types, we were unable to conduct enrichment or pathway analyses on all relevant metabolites, which may have resulted in missing some potential metabolite-LBP relationships. While MR analysis has assisted us in identifying CSF metabolites linked to LBP, prospective studies are still needed to further investigate their potential mechanisms.

## Conclusion

This two-sample MR study highlights the significant role of CSF metabolites in the risk of LBP. Twelve metabolites were found to be significantly causally associated with LBP, providing insight into the complex interactions between metabolic products and cerebrospinal fluid in the pathogenesis and progression of LBP. Moreover, metabolic biomarkers in cerebrospinal fluid can more directly reflect the condition of the central nervous system compared to blood metabolites, offering valuable research potential. These findings contribute to understanding the potential biological mechanisms of LBP and pave the way for future explorations of targeted therapeutic interventions.

## Electronic supplementary material

Below is the link to the electronic supplementary material.

Supplementary Material 1

Supplementary Material 2

Supplementary Material 3

## Data Availability

No datasets were generated or analysed during the current study.
