# Supplementary figures and images for: The causal effects between low back pain and cerebrospinal fluid metabolites: a two-sample Mendelian randomization study
Source: Hereditas. 2025 Feb 7;162:18. doi: 10.1186/s41065-025-00374-y (PMC11804052; doi:10.1186/s41065-025-00374-y)

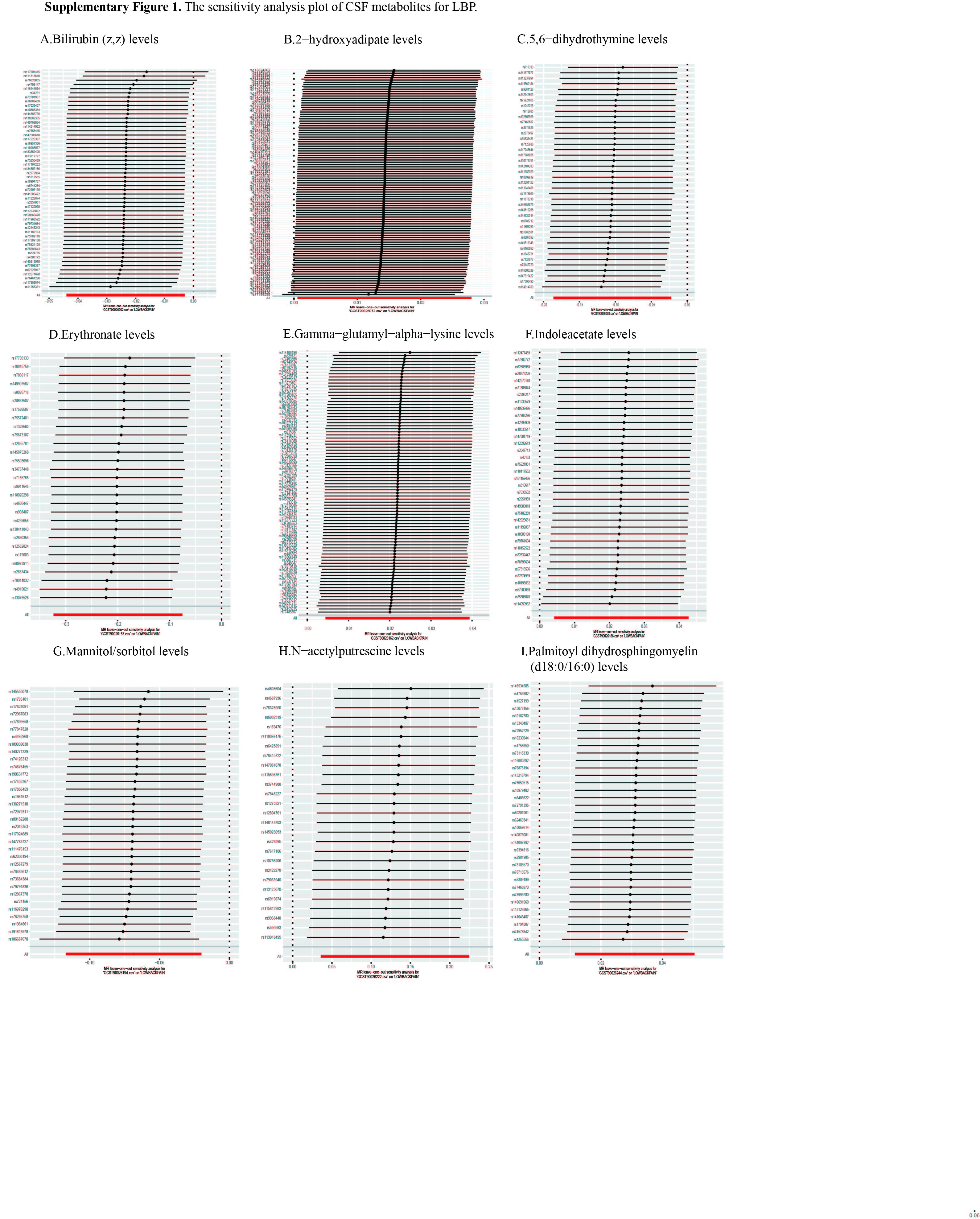

Supplement: Supplementary file 1 — Supplementary Material 1 [file 41065_2025_374_MOESM1_ESM.jpg]

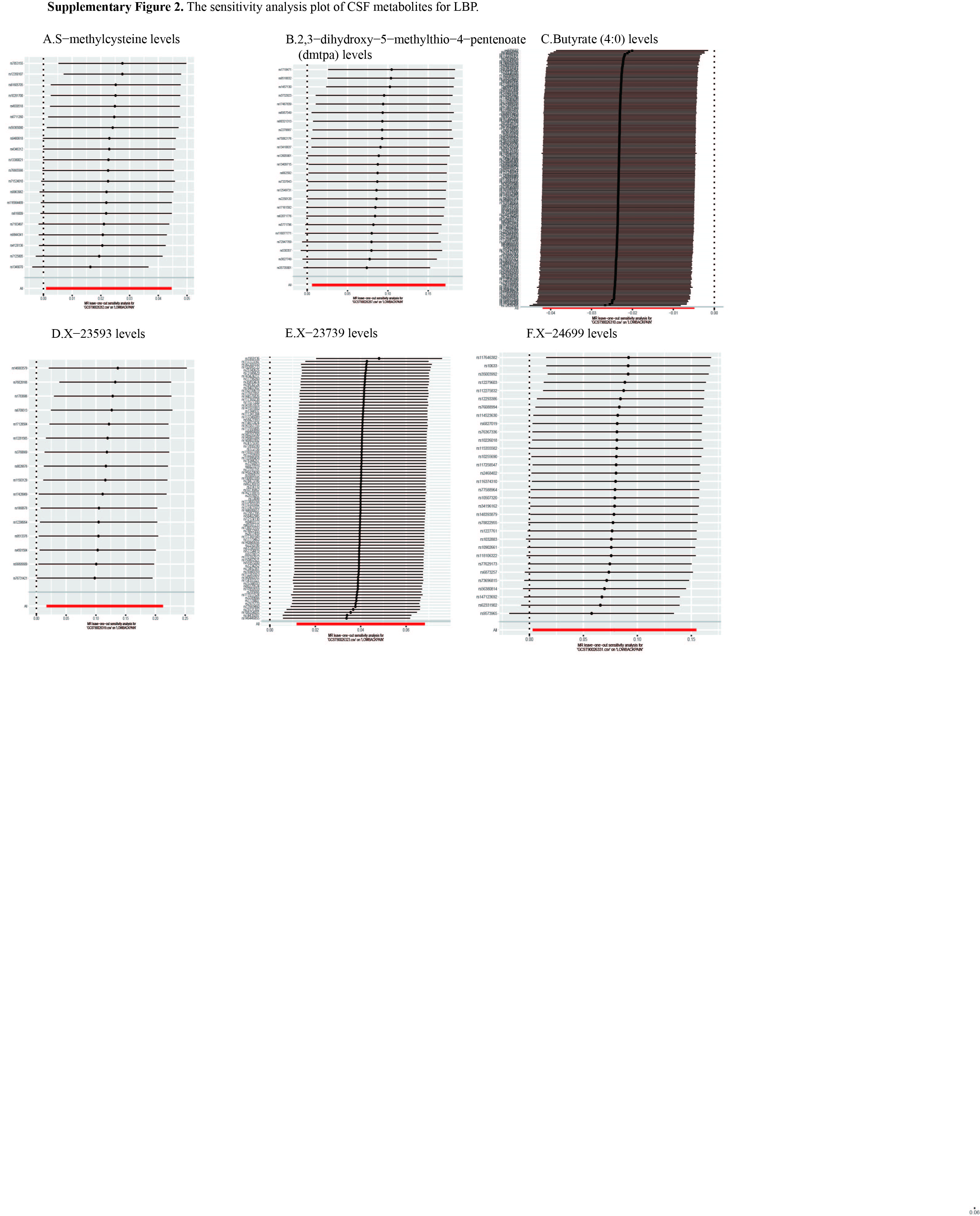

Supplement: Supplementary file 2 — Supplementary Material 2 [file 41065_2025_374_MOESM2_ESM.jpg]
