# Supplementary material for: The causal effects between low back pain and cerebrospinal fluid metabolites: a two-sample Mendelian randomization study
Source: Hereditas. 2025 Feb 7;162:18. doi: 10.1186/s41065-025-00374-y (PMC11804052; doi:10.1186/s41065-025-00374-y)
Supplement: Supplementary file 3 — Supplementary Material 3 [file 41065_2025_374_MOESM3_ESM.docx]

**Supplementary Table 1.** Data sources for used GWAS summary statistics.

| Category | Variable | Population | Resources | Sample Size | Year |
| --- | --- | --- | --- | --- | --- |
| Exposure | Cerebrospinal fluid metabolite | European | Panyard et al | 291 | 2017 |
| Outcome | Low back pain | European |  |  |  |
